# Compensatory Changes in the Anterior Segment and Vascular System of the Eye in Myopic Children After Orthokeratology

**DOI:** 10.3389/fped.2021.663644

**Published:** 2021-09-09

**Authors:** Yanwei Chen, Xi Shen

**Affiliations:** Department of Ophthalmology, School of Medicine, Ruijin Hospital Affiliated Shanghai Jiao Tong University, Shanghai, China

**Keywords:** orthokeratology, anterior segment, retina, vessel density, optcal coherenc tomography angiography

## Abstract

**Purpose:** To analyze changes in the anterior segment, retinal vessel density, and choroidal thickness (ChT) after orthokeratology (Ortho-K).

**Methods:** Myopic children were enrolled from Ruijin Hospital, Shanghai, China. Ortho-K lenses and single-vision spectacles were fitted for myopia correction. Ocular measurements were taken at baseline and 6 months, including axial length (AL), central corneal thickness (CCT), anterior chamber depth (ACD), lens thickness (LT), white to white (WTW), ChT, macular vessel density (MVD), and optic disc vessel density (OVD).

**Results:** Seventy-six patients were enrolled in this study, including 40 in the Ortho-K group and 36 in the control group. At baseline, no parameters between the two groups were statistically different. After 6 months, changes in CCT and ACD decreased in the Ortho-K group compared with those in the control group (*p* < 0.05); LT and ChT in the Ortho-K group were thicker than those in the control group (*p* < 0.05), while there was no difference in MVD and OVD compared with those in the control group (*p* > 0.05). There were moderate positive correlations between ChT and LT and between ChT and OVD in the Ortho-K group (*p* < 0.05).

**Conclusion:** The changes in the anterior and posterior segments of the eye after Ortho-K lens wearing suggest that the human eye has a powerful compensatory effect on the imposed defocus.

## Introduction

Myopia has been a problem in children and teenagers for decades, and the prevalence has increased worldwide, especially in East Asia (1). Myopia control has become a topic of interest to researchers. Studies demonstrate that children are less likely to be myopic when they spend more time outdoors (2). Orthokeratology (Ortho-K) has been proven to be effective in myopia control (3), and it also increases vision-related quality of life better than single-vision spectacles (4).

Ortho-K corrects myopia by changing the corneal formation (5, 6) and also changing the anterior segment structure (7, 8). However, previous articles have not explored the mechanisms of lens alteration in detail. Thickening of the choroid was also found after wearing the Ortho-K lenses using enhanced depth imaging (EDI) mode in optical coherence tomography (OCT).

OCT angiography (OCTA) is a noninvasive technology with potentially broad applicability for retinal vascular disease. It has been widely used in recent years for its deep scanning and retinal vascular quantitative analysis capabilities (9). Previous studies have proven that retinal microvasculature vanishes in high myopia patients (10–12). However, no study has reported whether the retinal vasculature changes after wearing Ortho-K lenses.

In the present study, in addition to changes in the anterior segment, we measured retinal vessel density and choroidal thickness (ChT) using OCTA to discover the effects of Ortho-K lenses on the posterior segment of the eye.

## Methods

### Study Design and Participants

This was a prospective, observational study. The design and procedure of this research adhered to the principles of the Declaration of Helsinki. The Ethics Committee of Ruijin Hospital authorized this study. Patients were selected from children diagnosed with myopia and treated with Ortho-K or single-vision spectacles at Ruijin Hospital from August 2019 to May 2020. Written informed consent was obtained from their parents.

The inclusion criteria were as follows: 8–14 years old, corrected distance visual acuity no less than 20/20, sphere between −1.00 D and −5.00 D, astigmatism less than −2.0 D, and no ophthalmologic or systematic disease. Enrolled participants had no treatment with other myopia control modalities, such as contact lenses or medicine. One eye was selected from each child for analysis.

Subjects in the Ortho-K group were fitted with a customized lens (Lucid, Seoul, Korea) by a specialized technician based on a standardized fitting criterion. The subjects were advised to wear the lenses for at least 8 h every night. Patients were revisited at 1 week, 1 month, 3 months, and 6 months. At each visit, the participants underwent a slit-lamp examination to check for any conditions that make their eyes unsuitable for Ortho-K lenses, such as severe corneal injury, allergy, and infection; if such conditions were found, the case was excluded.

In the control group, subjects were fitted with single-vision spectacles after cycloplegia. They were asked to revisit after 6 months.

### Clinical Examination Measurement

All participants underwent a complete ophthalmologic examination at baseline and each visit after cycloplegia. At baseline, all procedures were performed between 8 a.m. and 12 a.m.; at 6 months, subjects in the Ortho-K groups were asked to arrive at the hospital within 2 h after removing Ortho-K lenses, and subjects in the control group were examined before 12 a.m. Axial length [AL; defined as the distance from the anterior cornea to the retinal pigment epithelium (RPE)], central corneal thickness (CCT), anterior chamber depth (ACD; defined as the distance from the center of the corneal endothelial to the anterior surface of the lens capsule), lens thickness (LT), flat K (K1), steep K (K2), and white to white (WTW; defined as the external measurement of the distance from limbus to limbus in horizon direction) were measured with Lenstar (LS 900; Haag Streit AG, Koeniz, Switzerland). Three consecutive measurements were conducted from each subject at each measurement to determine the mean values.

**Figure 1:**
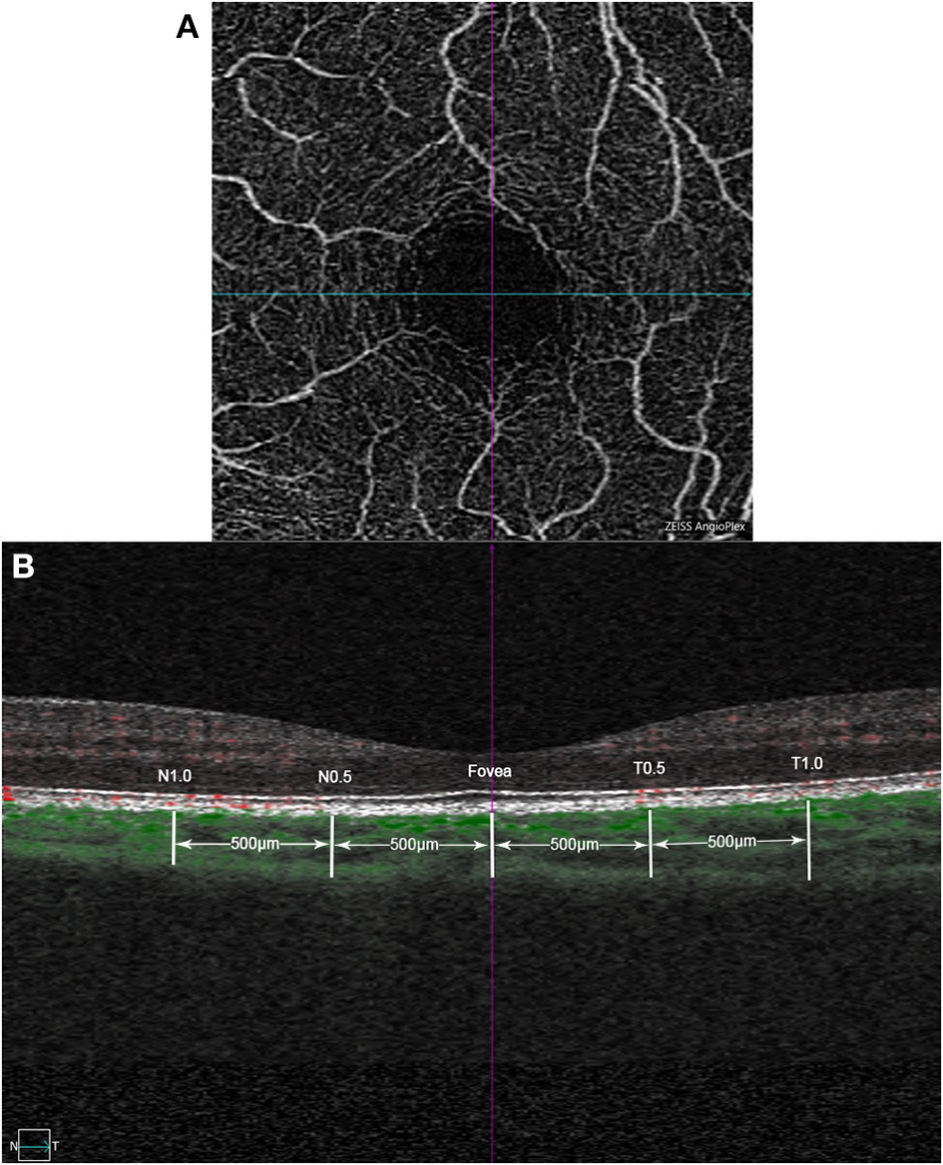
Example images of choroidal thickness measurement based on optical coherence tomography angiography (OCTA). **(A)** Example of macular superficial capillary plexus scan with angiography 3 × 3mm mode. The cross center is automatic located in the fovea by the built-in software of OCTA. **(B)** B-scan image at the same location, with the green part identified by the OCTA algorithm as the choroidal blood flow signal. Choroidal thickness (ChT) was manually measured in the horizontal direction, including points of fovea, 0.5 and 1.0mm from the fovea in the nasal and temporal directions, respectively.

OCTA scans were captured with Cirrus HD OCT 5000 (Carl Zeiss Meditec, Jena, Germany) software version 9.5.2. ChT was obtained with angiography in 3 × 3 mm EDI mode for a better detailed display. It contains 245 × 245 A-scans and 245 A-scans in each B-scan. ChT was defined as the distance from the hyperreflective line of Bruch's membrane to the line of the inner surface of the sclera. In the B-scan image, the choroidal blood flow signal is visualized as green by the OCTA algorithm, and the tomograph is automatically localized to the fovea. ChT was manually measured in the horizontal direction, including points of fovea, 0.5 and 1.0 mm from the fovea in the nasal and temporal directions, respectively. Each point was measured three times, and the average was obtained (Figure 1).

**Figure 2:**
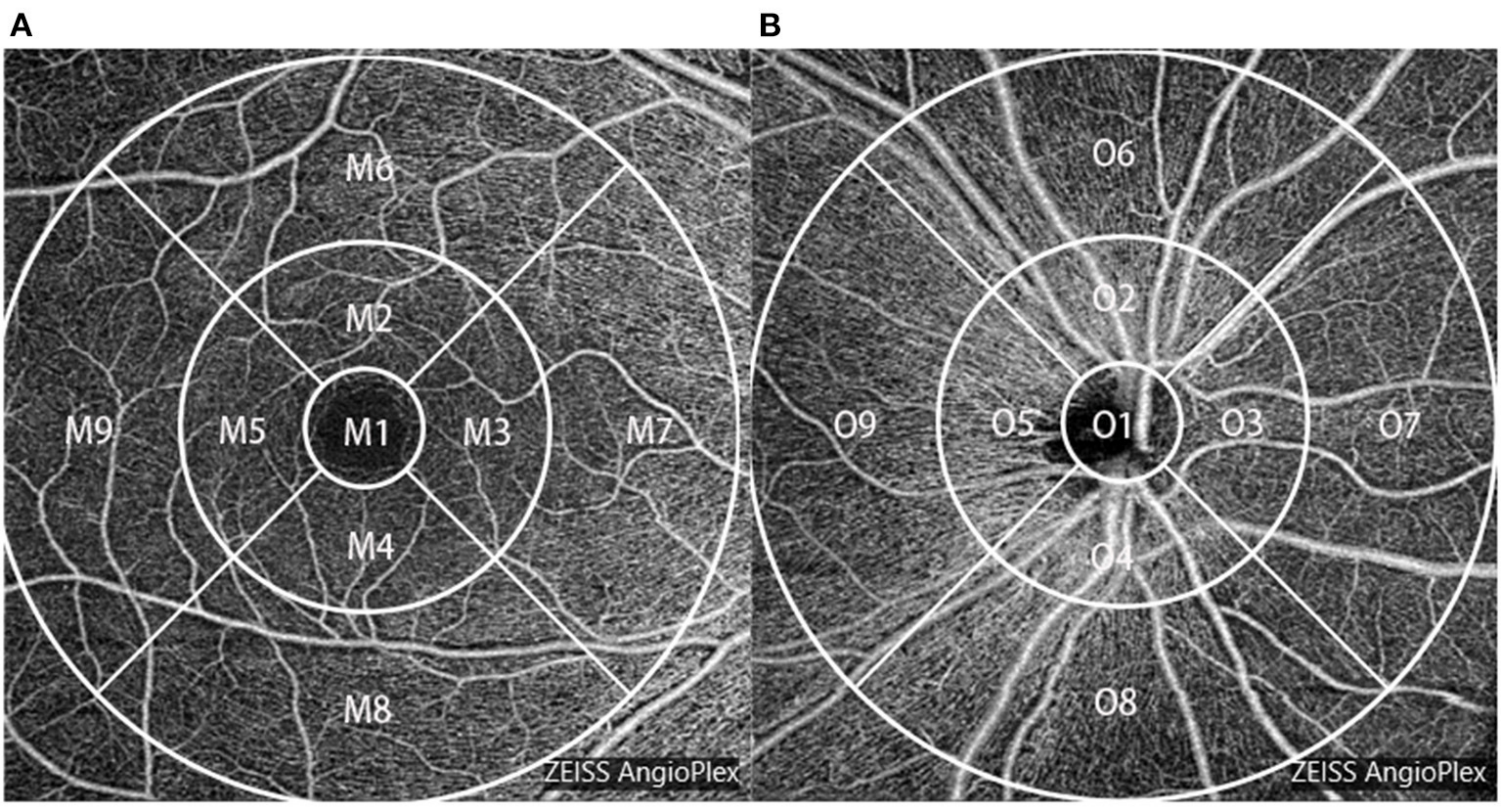
Vessel density map of the macula **(A)** and the optic disc **(B)** of the right eye based on optical coherence tomography angiography (OCTA).

The angiography 6 × 6 mm protocol was chosen to capture the superficial retinal vascular density. It contains 350 × 350 A-scans and 350 A-scans in each B-scan. The vessel density map of the macula and optic disc was a 6-mm-diameter circular area, divided into nine sections with three concentric rings according to the Early Treatment Diabetic Retinopathy Study (ETDRS) map. The inner ring was 1.0 mm in diameter, the middle ring was 3 mm, and the outer ring was 6 mm. The circle was centered on the fovea and optic disc; the software automatically calculated values in each region. Zones were recorded as M1–M9 and O1–O9 (Figure 2). Vessel density is the linear length of vessels divided by the selected dimension.

The Zeiss OCTA system is incorporated with a real-time retinal tracking technology named FastTrac^™^, which ensures consistency between pre- and post-scans.

One skilled doctor obtained all the OCT scans. Images with a signal higher than six and without noticeable artifacts were selected for analysis.

Manifest refractions were conducted at baseline, and the spherical equivalent refractive errors (SE = spherical degree + 0.5 × astigmatism degree) were recorded.

### Statistical Analysis

Statistical analysis was performed with SPSS 20.0 (IBM Corporation, Chicago, IL, USA). Data were tested for normality with the sample Kolmogorov–Smirnov (K-S) test. As most parameters of the anterior segment were non-normally distributed, the results are expressed as medians with interquartile intervals. Differences between the two groups were compared with the independent Student's *t*-test for normally distributed data and the Mann–Whitney *U* test for non-normally distributed data. A chi-square test was used to assess the difference in the female/male ratio between the Ortho-K group and the control. Correlations between ChT and LT and between ChT and retinal vessel densities were analyzed with Spearman's correlation coefficient. The results were considered statistically significant when *p* < 0.05.

## Results

Ultimately, a total of 76 children were eligible and completed the 6-month follow-up, including 40 in the Ortho-K group and 36 in the control group. The female/male ratio, mean age, SE, and baseline ocular parameters are shown in Table 1. There were no statistically significant differences between the two groups (*p* < 0.05).

**Table 1:** Demographic and biometric characteristics at baseline between the two groups.

	**Ortho-K group**	**Control**	***p***
Female/male	25/15	18/18	0.272
Age (year)	10.00 (9.00, 12.75)	10.00 (10.00, 12.00)	0.859
SE (D)	−2.50 (−3.00, −1.75)	−2.25 (−3.00, −1.50)	0.397
AL (mm)	24.83 (23.98, 25.31)	24.64 (24.30, 25.28)	0.727
CCT (μm)	555.50 (513.00, 574.75)	545.00 (530.75, 558.50)	0.141
ACD (mm)	3.30 (3.12, 3.44)	3.27 (3.10, 3.41)	0.632
LT (mm)	3.35 (3.29, 3.46)	3.34 (3.23, 3.49)	0.365
K1 (D)	42.65 (41.28, 44.05)	41.80 (41.31, 42.92)	0.151
K2 (D)	43.89 (42.02, 45.36)	43.26 (42.53, 44.43)	0.315
WTW (mm)	12.04 (11.68, 12.34)	12.19 (11.85, 12.35)	0.336

*Ortho-K, orthokeratology; SE, spherical equivalent; AL, axial length; CCT, central corneal thickness; ACD, anterior chamber depth; LT, lens thickness; WTW, white to white*.

After 6 months, the changes in the AL, CCT, ACD, K1, and K2 of the Ortho-K group were smaller than those in the control group, while the change in the LT was more significant than that in the control group. The WTW between the two groups was not significantly different at 6 months (Table 2).

**Table 2:** Mean changes from baseline in ocular parameters during 6 months of follow-up in the Ortho-K group and the control group.

	**Ortho-K group**	**Control**	***p***
AL (mm)	0.03 (−0.01, 0.10)	0.14 (0.10, 0.25)	<0.000
CCT (μm)	−8.00 (−16.00, −3.25)	1.50 (−1.00, 6.75)	<0.000
ACD (mm)	−0.04 (−0.08, −0.01)	0.00 (−0.02, 0.03)	<0.000
LT (mm)	0.04 (0.02, 0.07)	0.00 (−0.02, 0.03)	<0.000
K1 (D)	−2.42 (−3.20, −1.70)	−0.05 (−0.18, 0.26)	<0.000
K2 (D)	−2.24 (−3.66, −1.77)	0.01 (−0.15, 0.30)	<0.000
WTW (mm)	−0.03 (−0.20, 0.05)	0.01 (−0.07, 0.12)	0.073

*Ortho-K, orthokeratology; AL, axial length; CCT, central corneal thickness; ACD, anterior chamber depth; LT, lens thickness; WTW, white to white*.

**Figure 3:**
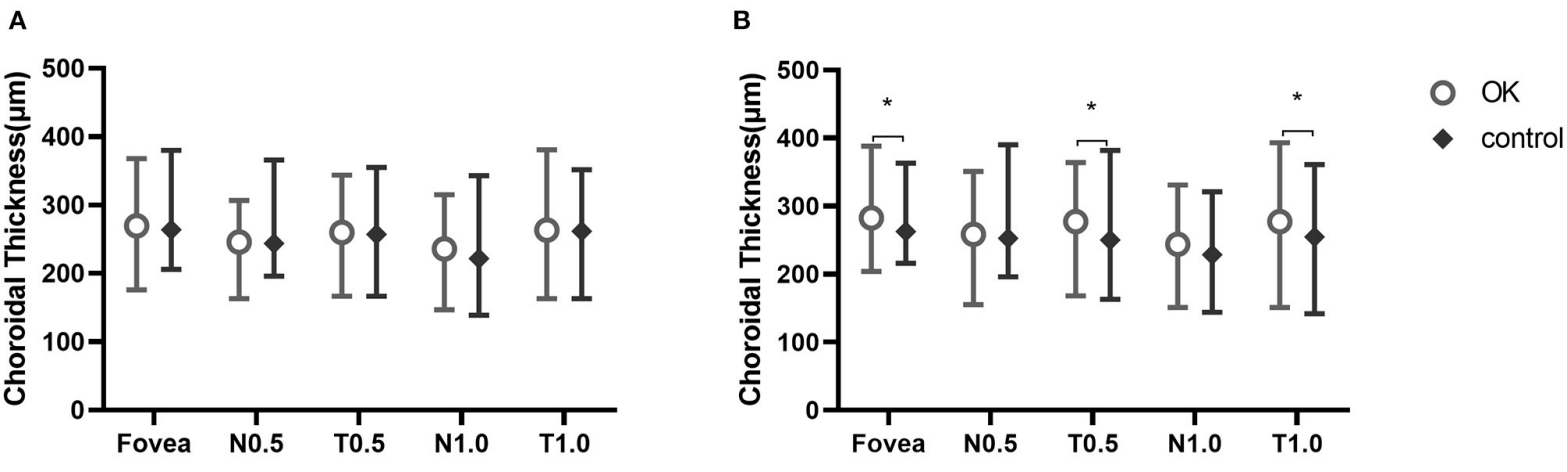
Choroidal thickness at five measurement points at baseline **(A)** and at 6 months **(B)**. ^*^Statistically meaningful.

ChT was not different at baseline between the two groups. After 6 months, ChT was thicker at the fovea and at T0.5 and T1.0 in the Ortho-K group than in the control group (Figure 3).

**Figure 4:**
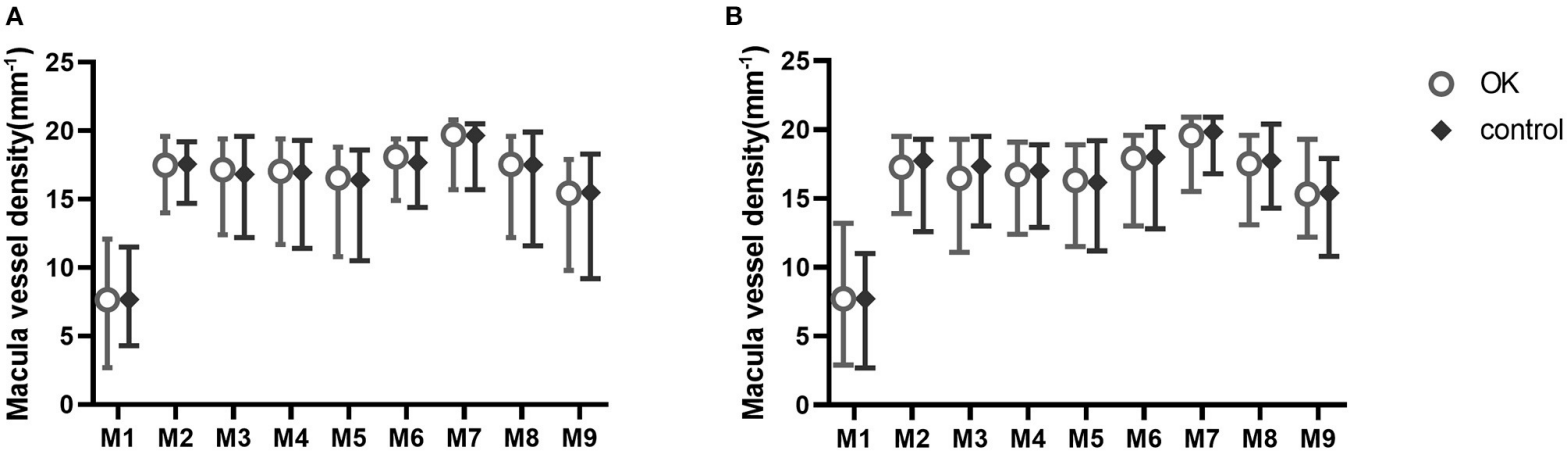
Nine zones of macular vessel density at baseline **(A)** and at 6 months **(B)**.

**Figure 5:**
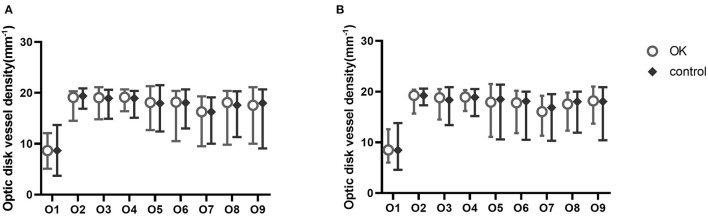
Nine zones of optic disc vessel density at baseline **(A)** and at 6 months **(B)**.

The retinal vessel densities in nine regions of the macula and optic disc were not significantly different at baseline or 6 months (Figures 4, 5).

**Figure 6:**
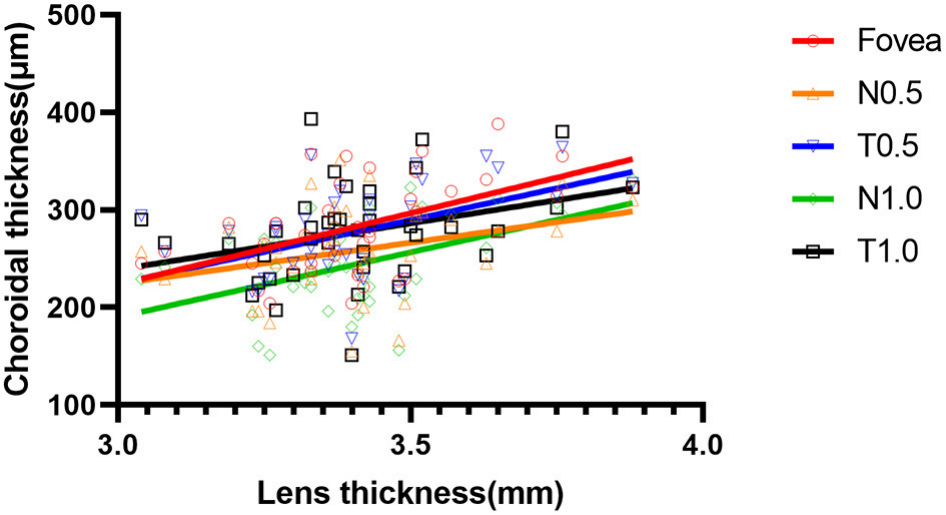
Scatter plot of the correlation between lens thickness and choroidal thickness at five measurement points at 6 months of the orthokeratology (Ortho-K) group.

At 6 months, there were positive correlations between LT and ChT at the fovea and at N0.5, T0.5, N1.0, and T1.0 in the Ortho-K group (*r* = 0.493, *p* = 0.001; *r* = 0.337, *p* = 0.034; *r* = 0.502, *p* < 0.001; *r* = 0.397, *p* = 0.011; and *r* = 0.352, *p* = 0.026, respectively) (Figure 6).

**Figure 7:**
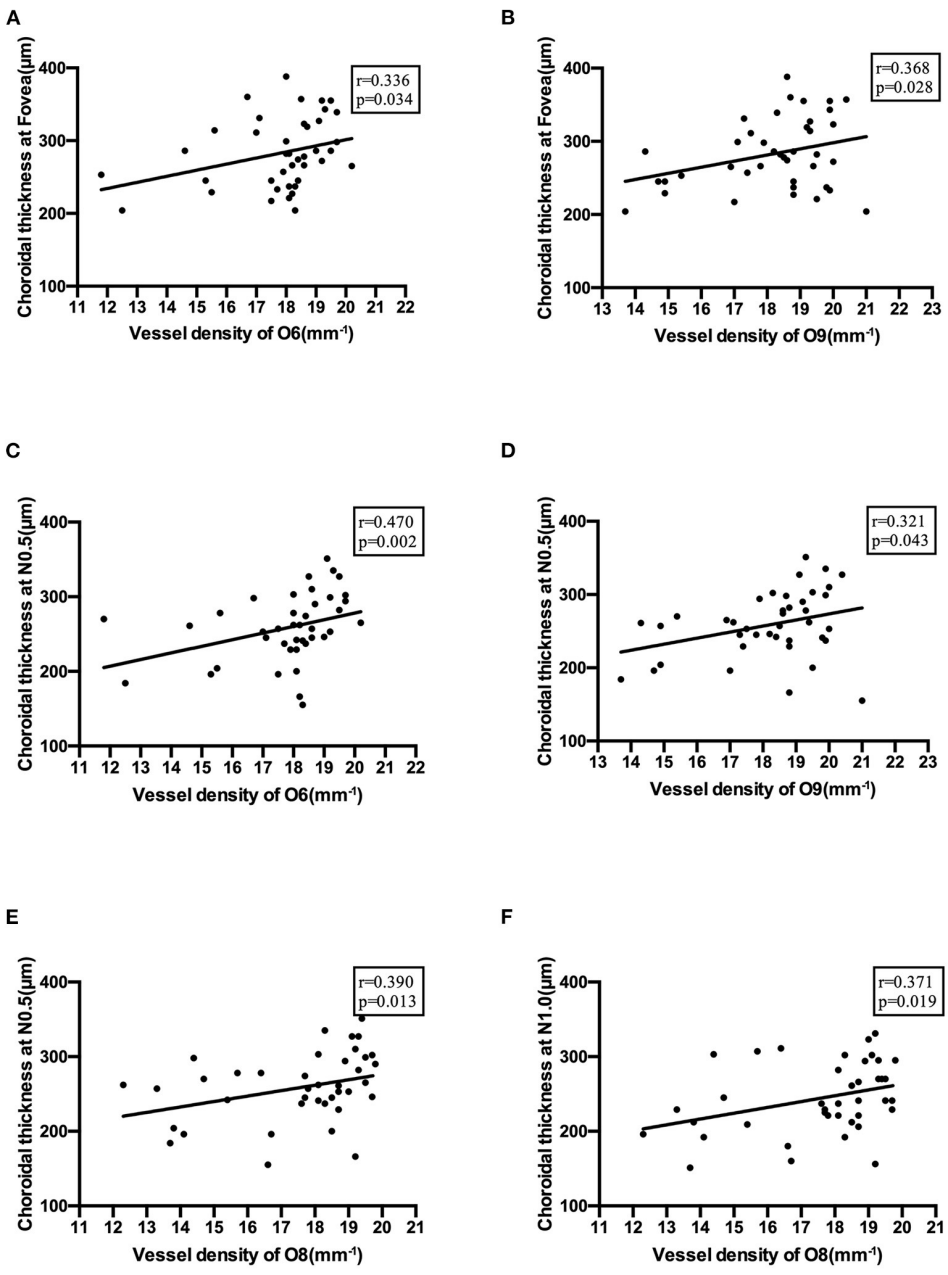
Correlations between choroidal thickness and vessel density in the optic disc area. **(A)** Correlation of choroidal thickness at the fovea with vessel density in O6 section. **(B)** Correlation of choroidal thickness at the fovea with vessel density in O9 section. **(C)** Correlation of choroidal thickness at N0.5 with vessel density in O6 section. **(D)** Correlation of choroidal thickness at N0.5 with vessel density in O9 section. **(E)** Correlation of choroidal thickness at N0.5 with vessel density in O8 section. **(F)** Correlation of choroidal thickness at N1.0 with vessel density in O8 section.

ChT also showed moderate positive correlations with vessel density in the optic disc area (Figure 7).

## Discussion

Consistent with the results of numerous previous studies, our study found choroidal thickening in myopic children who wore Ortho-K lenses. The mechanism of choroidal thickening is thought to be primarily due to peripheral myopic defocus caused by Ortho-K lens wear, which is one of the mechanisms controlling myopia growth. As early as the 1990s, Josh Wallman determined through a series of experiments that the choroid can respond rapidly to lens-induced defocus by modulating the focal plane of the retina to the eye through thickness changes (choroidal accommodation) (13). Imposed defocus can produce rapid changes in ChT in humans within 60 min of lens wear (14). Myopic defocus slowed axial elongation more effectively when it was confined to the peripheral retina rather than when the same amount of myopic defocus was projected onto the entire retina (15). Hemifield myopia defocus can lead to a local regional choroidal response (16), and patients with presbyopia can also observe choroid thickening by myopic defocus (17). Previous studies have suggested five possible mechanisms that may explain why the choroid increased rapidly: (1) increased synthesis of large, osmotically active proteoglycans pulled water into the choroid; (2) the size or amount of fenestrations in the choriocapillaris had increased, which increased the amount of osmotically active molecules in the choroid; (3) the aqueous humor entered the choroid as part of the drainage of fluid; (4) the transport of fluid from the retina across the RPE was altered; and (5) the tonus of the non-vascular smooth muscle changed. It is likely that more than one of these mechanisms is involved (13). In our study, we found thickening in only the fovea and the temporal part of the ChT, but not the nasal part of the ChT. Because the nasal choroid itself is thinner (18), it may have less potential for thickening than that in the fovea and the temporal part.

In addition to choroidal thickening, the lens was also thickened in our study. The same finding was mentioned in a previous study, but the mechanism was not explored in detail (7, 8). Some investigators have suggested that this is due to regulation to compensate for the small amount of overcorrection caused by Ortho-K lens correction (19), but thickening is still detected after ciliary muscle paralysis (7, 20). Myopic patients wearing Ortho-K lenses have increased accommodation amplitude and improved accommodation hysteresis (21, 22), and these changes may be a cause rather than a consequence of lens thickening.

In addition to its accommodation function, the lens also compensates for corneal aberrations. After wearing Ortho-K lenses, the high-order aberration (HOA) of the eye increases due to changes in corneal morphology; the HOA is regarded as associated with the control of myopia progression (23). There was a more significant increase in corneal rather than ocular spherical aberrations (SAs), indicating that changes in internal SAs are mainly generated by the lens (24, 25).

In a study of lens power in children and adolescents, the authors found a negative association between lens power and AL, and it was more evident in nonmyopia than in myopia. This finding may indicate that myopia develops when the lens power cannot compensate for axial growth (26, 27).

In a chick model, researchers found that lens size and shape were not altered after the induction of refractive errors (28). In contrast, other studies found that the lens focal lengths were longer than those of the controls, and accommodative changes were more significant when eyes were imposed with +15 D blur (29). In addition, the results suggested that the myopic and hyperopic lens treatments had different influences on lens delta-crystalline formation (30).

Some researchers believe that the shape and location of the lens change to compensate for some of the aberrations of the cornea (31), or the lens is genetically preprogrammed to respond to specific putative retinal factors and is capable of identifying and regulating its growth changes (29).

Previous studies have reported that only CCT was reduced after long-term use of the Ortho-K lens, while the ACD, LT, and anterior segment length were maintained (20, 32). Other researchers found that ACD was reduced after Ortho-K lens wearing (7), even in adults (6). In this study, we believed that a thickened lens might lead to decreased ACD.

The correlation between LT and ChT may suggest that in addition to alterations in the lens itself, the ciliary body's anatomical association with the choroid suggests that the ciliary body may also be involved in the alteration of the lens shape. We speculated that a thickened lens led to a smaller anterior chamber volume and that extra humor fluid entered the choroid, thickening the ChT.

Researchers have used OCTA to detect the variation of vessel density of the retina in different degree myopia patients and longer AL associated with less superficial retinal VD in adults (11, 12). However, in children, varying results have been reported in different studies (33–35). The distribution of age and AL may explain the differences in the results. In this study, we followed up for only 6 months to minimize the effect of increasing AL on vessel density measurements.

The retina has both the retinal vasculature supplying the inner retinal layers and the choroidal vascular bed supplying the outer retinal layers, which is the most metabolically active layer. However, there is no clear boundary between the two blood circulations (36). Because of the high metabolic activity of the retina, the ability to accommodate blood flow is an important feature of the mammalian retina due to its high metabolic activity (37).

The retina can respond to changes in defocus and aberration, which occur at the photoreceptor level (38). We speculated that metabolic feedback mechanisms might mediate blood flow regulation in response to changes in neuronal activity. Increasing neuronal activity leads to high metabolism and lower O_2_ and glucose levels and produces vasoactive metabolites. These metabolites elicit vasodilation and increase blood flow (37). Nitric oxide (NO) might be one of the meditators regulating blood flow. NO was found to contribute to blood flow regulation in the retina by modulating the glia-to-vessel signal path (39). Increases in choroidal blood flow are regulated by parasympathetic efferent nerves, which also function through the NO signaling pathway (40). These regulatory mechanisms may explain the correlation between ChT and retinal vessel density in this study.

The main limitations of this paper are the small sample size, the failure to observe ocular changes after Ortho-K lenses cessation, and the failure to correct the magnification of the OCT scans.

The human eye is a sophisticated structure. This study showed that in response to imposed optical defocus, the anterior and posterior segments of the eye compensate for these changes. These results may reveal new mechanisms underlying the slowing down of myopia progression by Ortho-K lenses.

## Data Availability Statement

The raw data supporting the conclusions of this article will be made available by the authors, without undue reservation.

## Ethics Statement

The studies involving human participants were reviewed and approved by The Institutional Review Board of Ruijin Hospital. Written informed consent to participate in this study was provided by the participants' legal guardian/next of kin.

## Author Contributions

YC recruited the patients, analyzed the data, and wrote the manuscript. XS performed a critical revision of the manuscript for intellectual content. All authors have read and approved the final manuscript, conceived and designed the protocol.

## Conflict of Interest

The authors declare that the research was conducted in the absence of any commercial or financial relationships that could be construed as a potential conflict of interest.

## Publisher's Note

All claims expressed in this article are solely those of the authors and do not necessarily represent those of their affiliated organizations, or those of the publisher, the editors and the reviewers. Any product that may be evaluated in this article, or claim that may be made by its manufacturer, is not guaranteed or endorsed by the publisher.
